# Telehealth Acceptance and Perceived Barriers Among Health Professionals: Pre-Post Evaluation of a Web-Based Telehealth Course

**DOI:** 10.2196/74107

**Published:** 2025-09-03

**Authors:** Lena Rettinger, Lukas Maul, Peter Putz, Veronika Ertelt-Bach, Andreas Huber, Susanne Maria Javorszky, Elisabeth Kupka-Klepsch, Sevan Sargis, Franz Werner, Klaus Widhalm, Rosmarie Joseph, Stefanie Doci, Sebastian Kuhn

**Affiliations:** 1 Department Health Sciences, FH Campus Wien, University of Applied Sciences, Favoritenstrasse 232, Vienna, 1100, Austria, 43 1 606 68 77 ext 4382; 2 Institute of Digital Medicine, Philipps-University and University Hospital of Giessen and Marburg, Marburg, Germany; 3 Department Applied Nursing Sciences, FH Campus Wien, University of Applied Sciences, Vienna, Austria

**Keywords:** telehealth, digital health, remote care, health care professionals, education, barriers, acceptance, web-based training

## Abstract

**Background:**

The rapid expansion of telehealth underscores the need for comprehensive telehealth education among health care professionals. Despite increasing recognition of telehealth’s importance, many practitioners remain underprepared, particularly in navigating legal aspects, technology, and patient engagement.

**Objective:**

This study aimed to evaluate the impact of a web-based telehealth training course on health care professionals’ telehealth acceptance and their perceived barriers to telehealth adoption.

**Methods:**

An interventional study with a pre-post design was used in Austria. A total of 365 health professionals enrolled in an asynchronous web-based course covering general telehealth principles (concepts, legal and technical aspects, practical implementation) and profession-specific content (eg, nursing, speech therapy, and physiotherapy). Of these, 217 completed the course, and 185 met inclusion criteria for analysis. Participants’ telehealth acceptance (covering telemetry, telephasis, and telepraxis) and perceived barriers were assessed via standardized questionnaires before and after the course. Satisfaction with the training was measured post-intervention using the Training Evaluation Inventory. Qualitative insights were gathered from open-ended survey questions and 2 focus groups, transcribed, and summarized.

**Results:**

Post-intervention, overall telehealth acceptance increased significantly (*P*<.001, *r*=0.21), particularly for telemetry (remote assessment and monitoring), telepraxis (remote interventions), video call–based, and asynchronous telehealth. Perceived barriers to telehealth use—such as uncertainty about legal frameworks, data protection, and reduced quality of care—diminished significantly (*P*<.001, *r*=0.39). Post-intervention satisfaction was high, with a total median Training Evaluation Inventory score of 76 (IQR 13). Participants rated the course highly for its clarity, breadth of content, and inclusion of profession-specific modules. Qualitative feedback highlighted a desire for more hands-on demonstrations, interactive components, and guidance on institutional support and patient accessibility.

**Conclusions:**

A structured, on-demand telehealth course significantly improved health professionals’ awareness, acceptance, and intention to use telehealth and reduced perceived barriers. While the findings highlight that targeted web-based training can increase clinicians’ confidence and readiness to use telehealth, it remains uncertain whether this will lead to an increase in its utilization. Future initiatives should incorporate blended-learning formats with additional practical examples, real-time discussions, and ongoing support to enhance long-term integration of telehealth into clinical workflows. On a policy level, we suggest coordinated actions at the EU, national, and institutional levels to standardize telehealth education and facilitate its practical implementation in everyday clinical practice.

## Introduction

### Background

The rapid adoption of telehealth technologies has underscored the necessity for adequate training among health professionals. Telehealth education is increasingly recognized as essential in preparing health care providers to deliver remote care effectively [1]. However, inconsistencies in integrating telehealth into curricula have been identified, along with a notable lack of formal study designs assessing telehealth education [2].

A significant barrier to the adoption of telehealth among providers is the deficiency of specific education and training [2,3]. For instance, graduate nursing programs often lack adequate telehealth preparation, leaving future nurses unprepared for remote patient care [4]. Similarly, allied health curricula exhibit substantial educational deficits in telehealth training, indicating an urgent need for primary research to enhance the quality and consistency of telehealth education [5].

Students acknowledge the significance of telehealth in their future professions but report low levels of knowledge and experience with telehealth applications. This gap highlights the importance of equipping students with the necessary competencies in a rapidly evolving health care landscape [6]. Training initiatives in specific disciplines, such as occupational therapy and physical therapy, have shown potential in enhancing telehealth usage [7,8]. Those have been demonstrated to improve confidence and proficiency in telehealth among practitioners [7]. However, existing telehealth training programs often are either not specifically developed for certain professions or fail to address all the unique capabilities required for remote care delivery [8]. This limitation may hinder the effective adoption of telehealth practices.

Despite these challenges, outcomes from telehealth education and training studies demonstrate high student satisfaction, with many expressing a willingness to incorporate telehealth into their future practice. Improvements in students’ problem-solving skills, acceptance of technology in patient care [2,9], improved telehealth knowledge and confidence [9,10], and enhanced telehealth and communication competencies [1,4,11] have also been noted. Moreover, it fosters a belief that they will use these skills in future practice [12]. Web-based learning tailored to learners’ needs has the potential to change knowledge, attitudes, and willingness to incorporate telehealth into routine care [13].

Despite the recognized importance of structured telehealth education and the positive outcomes associated with such training, there remains a lack of formal study designs evaluating its impact on health care professionals’ acceptance and perceived barriers. Moreover, there is a scarcity of educational offerings for already qualified health care professionals who did not receive telehealth education during their initial training. This gap leaves many practicing clinicians without the necessary skills and knowledge to adopt telehealth technologies effectively.

### Aim

Therefore, this study aimed to evaluate the impact of a web-based telehealth training course on health care professionals’ acceptance of telehealth and the perceived barriers to its adoption. By using a pre-post design, the study seeks to provide insights that can contribute to the improvement of telehealth education and promote the integration of telehealth into clinical practice.

## Methods

### Study Design

This study used a pre-post design to evaluate the impact of an interprofessional web-based telehealth training course on health care professionals’ acceptance of telehealth and perceived barriers to its adoption.

### Participants

Participants in this study were health care professionals. Inclusion criteria required participants to be physiotherapists, occupational therapists, orthoptists, nurses, speech and language therapists, or dietitians. Professionals from other health care fields were also eligible to participate in the course and study, but the course content was specified for the aforementioned professions.

### Procedure

Participants were recruited through professional associations of the targeted professional groups. Interested individuals were invited to register via a web-based form. On May 1, 2024, a participation link was emailed to all registered participants, and they were able to participate in the study until August 31, 2024. As a measure to increase the response rate, 2 reminder emails were sent to registered participants on August 8 and August 26, 2024.

The web-based training as well as the pre- and post-intervention questionnaires were administered using the web-based survey platform “LimeSurvey.” Participants completed the initial questionnaire prior to commencing the web-based course and filled out the follow-up questionnaire upon completion of the training. Participants were able to pause their participation at any time during the study.

### Ethical Considerations

The survey followed ethical research practices as outlined in the Declaration of Helsinki, ie, voluntary participation; assurance of anonymity, data protection, and confidentiality; advance information about the purpose and content; provision of contact information; and disclosure of the organization conducting the research and the funding source. In accordance with the regulations of the Ethical Committee at FH Campus Wien, assessments involving non–health-related data from non-vulnerable persons do not require formal ethical approval. All participants received written information before participation. By completing the questionnaire, participants provided informed consent for the anonymous use of their data for research and publication purposes. Participation was entirely voluntary, and participants could pause or withdraw at any time without consequences. Participants received continuing education credits upon completion of the course.

### Intervention: Telehealth Training Course

**Figure 1.**
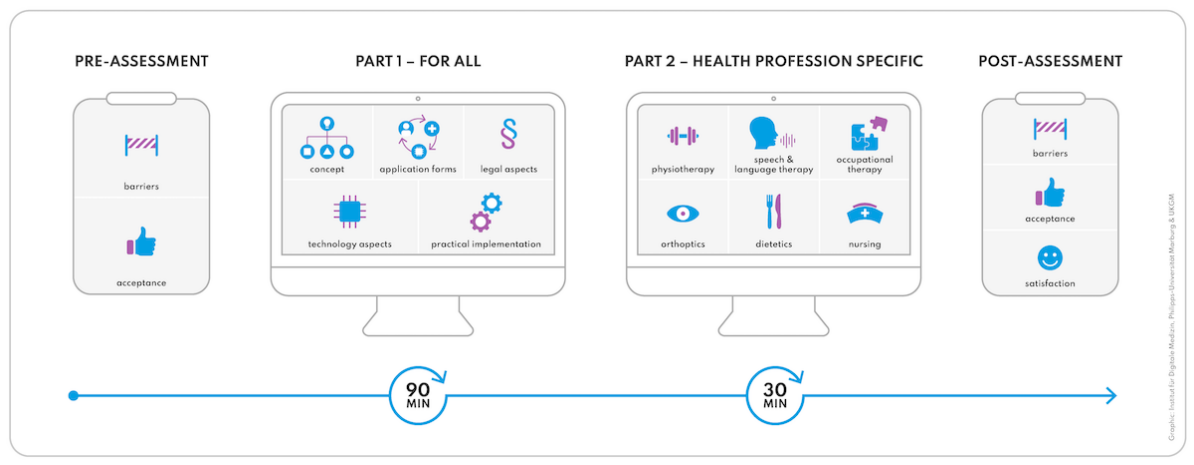
Structure of the assessments and the telehealth training course.

The web-based telehealth course was structured into a general and profession-specific video format (Figure 1). The general part of the course, which all participants were required to complete, consisted of the following videos: concept clarification (6:00 min), application forms (18:07 min), legal aspects (18:44 min), technology aspects (18:01 min), and practical implementation (30:20 min). Following the general part, participants were asked to watch a video tailored to their specific profession with a duration of approximately 30 minutes.

Participants from other professions were able to choose one of the specialized videos that best matched their area of practice. A detailed summary of the video content can be found in Multimedia Appendix 1.

### Data Collection

Before watching the training videos, participants were asked to complete a demographic survey to collect data such as age, gender, profession, current employment status, years of experience, primary client group, federal state, population density, perceived patient access (based on health profession), and general and specific telehealth experience. Data on acceptance (willingness to adopt) and perceived influence of telehealth barriers were also collected both before and after the intervention using standardized scales. Finally, the participants rated their satisfaction with the course.

#### Telehealth Experience and Acceptance

**Figure 2.**
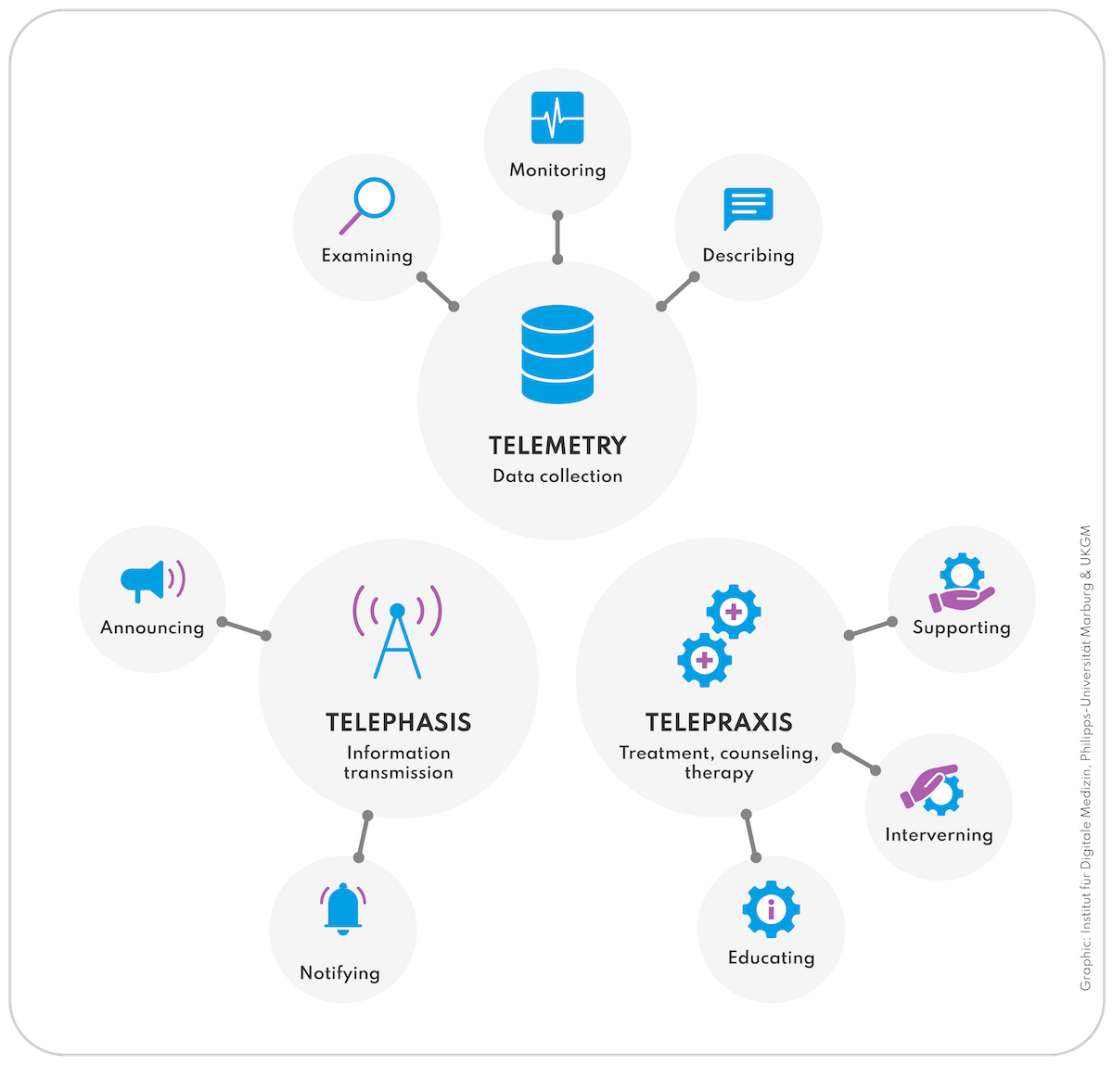
Visual representation of the classification system based on Colucci et al (2019) to assess specific telehealth experience.

We measured specific telehealth experiences and acceptance using a classification system based on the work of Colucci et al [14]. This system defines telehealth according to its communication processes, including telemetry, telephasis, and telepraxis (Table 1 and Figure 2). Participants self-reported whether they had experienced each telehealth application via phone, video calls, or asynchronously (yes or no). They also indicated their willingness to adopt each telehealth process through these modes, using a Likert scale from 0 (not at all willing) to 4 (fully willing).

**Table 1.** Adapted classification system based on Colucci et al (2019) to assess specific telehealth experience and acceptance of the participants.

Function and application	Description or example
Telemetry	
Data collection	
Examining	Obtaining findings, performing measurements
Monitoring	Monitoring of vital data, movement, or activity behavior
Describing	Verbally or in writing, recording the client’s medical history
Telephasis	
Information transmission	
Announcing	Public dissemination of health information, eg, on blogs, videos, websites
Notifying	Individual forwarding of information; eg, about findings, measurement results
Telepraxis	
Treatment, counseling, and therapy	
Supporting	eg, implementing therapy recommendations at home, providing environmental support
Intervening	eg, direct treatment or therapy
Educating	eg, health education, informing about lifestyle modifications

#### Perceived Influence of Barriers

The barriers were measured based on a framework developed by Rettinger and Kuhn [3]. Participants rated the extent to which each of the 24 barriers influenced their willingness to adopt telehealth, using a Likert scale ranging from 0 (no influence) to 4 (strong influence).

#### Satisfaction With the Course

After the course, participants completed the Training Evaluation Inventory [15] to assess their satisfaction with the course content, subjective enjoyment, perceived usefulness, perceived difficulty, subjective knowledge gain, and attitude towards training. Additionally, they were asked for feedback in their own words. In September 2024, two focus groups were held with participants who volunteered to discuss the course in more detail.

### Data Analysis

Participants who engaged with at least 40 minutes of the total 120-minute video content were included in the analysis; this threshold was established to ensure sufficient exposure to the intervention. Participants not meeting this criterion were excluded from subsequent analyses. Demographic data were summarized using means and standard deviations for continuous variables and frequencies and percentages for categorical variables.

Sum scores for telehealth acceptance were calculated: total telehealth acceptance (max 96), telemetry acceptance (max 48), telephasis acceptance (max 24), telepraxis acceptance (max 48), acceptance of telehealth via phone (max 32), acceptance of telehealth via video call (max 32), and acceptance of asynchronous telehealth (max 32). A total barrier score was calculated also with a sum-score (max 96). Acceptance of telehealth processes and perceived barriers, both before and after the intervention, were analyzed using medians and interquartile ranges (IQRs) due to the ordinal nature of the data. The Wilcoxon signed-rank test was used to assess differences between pre- and post-intervention scores, with the significance level set at alpha=.05. This test evaluates differences in the distribution of repeated-measures data by calculating the sum of the ranks. This test can reveal significant differences between repeated measures, even when their medians are equal. Effect sizes were calculated using the r statistic, calculated as z/sqrt(n), and interpreted according to Hattie’s guidelines [16,17], where *r*<0 indicates a negative effect, *r*=0.00 indicates no effect (developmental effects), *r*=0.05 indicates a small effect (teacher effects), *r*=0.10 indicates a medium effect (zone of desired effects), and *r* ≥ 0.15 indicates a large effect, thereby providing context for the magnitude of the findings.

Satisfaction with the web-based course was evaluated post-intervention and presented as medians and IQRs. These satisfaction scores were further analyzed by professional groups to identify any differences across professions.

Qualitative data were obtained from 2 focus groups and written feedback collected through questionnaires. The focus group discussions were transcribed using MAXQDA AI-Assist. The transcribed and anonymized texts were then summarized using ChatGPT (version o1-preview). To ensure the validity of these summaries, the first 2 authors—who also moderated the focus groups—reviewed the summarized results for accuracy and completeness, adjusting where needed.

#### Sensitivity Analysis for Non-Representativeness of the Sample

When comparing the occupational and gender distribution of the sample to the population, male participants and participants from the nursing profession were found to be severely overrepresented. Therefore, an additional sensitivity analysis was conducted to take non-representativeness into account by case weighing. In this procedure, all data related to inferential statistics were post-harmonized on the basis of known population distributions, available from the annual report of the Austrian Register of Health Professions [18]. For this purpose, the following weighting factors (as shown in parentheses) were calculated for four strata: (1) female therapists (0.19), (2) male therapists (0.43), (3) female nurses (8.81), and (4) male nurses (3.48). Physiotherapists, occupational therapists, orthoptists, and speech and language therapists were thereby combined under the category “therapists.” Weighting factors were calculated by dividing the target stratum proportion by the actual stratum proportion. The post-harmonized data can thus be considered representative of occupation (therapists vs nurses) and gender (female vs male). However, none of these post-harmonized inferential analyses (data not shown) differed from the unadjusted analyses in terms of statistical significance and effect interpretation, and therefore only the unadjusted results are reported in the results section.

## Results

### Participants

A total of 365 health professionals enrolled in the web-based course. Of these, 217 participants completed the course. Among the completers, 185 were included in the analysis: 63 physiotherapists (34%), 48 occupational therapists (26%), 38 orthoptists (21%), 20 nurses (11%), 9 speech and language therapists (5%), and 7 other health professionals (dietitian, diabetes counselor, midwife, psychologist, paramedic in training, student, and administrator). Mean engagement with the video content was 198 minutes (minimum 40; maximum 3015). In total, 32 course completers were excluded from analysis for not meeting the 40-minute minimum engagement threshold. The demographic characteristics of the participants are summarized in Table 2.

**Table 2.** Participants’ characteristics.

Characteristics	PT^a^ (n=63)	OT^b^ (n=48)	ORT^c^ (n=38)	NUR^d^ (n=20)	SLT^e^ (n=9)	Other (n=7)	Total (n=185)
Age, mean (SD), years	40.38 (10.57)	35.94 (9.58)	43.13 (11.48)	44.05 (8.63)	39.89 (9.21)	37.43 (10.01)	40.05 (10.52)
Professional experience in years, mean (SD)	16.02 (11.44)	11.73 (9.67)	19.84 (12.79)	20.25 (10.53)	15.33 (9.07)	15.98 (11.39)	15.98 (11.39)
Gender, n (%)							
Female	51 (80.95)	46 (95.83)	36 (94.74)	14 (70.00)	9 (100.00)	6 (85.71)	162 (87.57)
Male	12 (19.05)	2 (4.17)	2 (5.26)	6 (30.00)	0 (0.00)	1 (14.29)	23 (12.43)
Primary client group, n (%)							
Children and youth	7 (11.11)	9 (18.75)	21 (55.26)	1 (5.00)	5 (55.56)	0 (0.00)	43 (23.24)
Adults	35 (55.56)	31 (64.58)	11 (28.95)	14 (70.00)	4 (44.44)	5 (71.43)	100 (54.05)
Seniors	21 (33.33)	8 (16.67)	6 (15.79)	5 (25.00)	0 (0.00)	2 (28.57)	42 (22.70)
Federal state, n (%)							
Burgenland	1 (1.59)	1 (2.08)	4 (10.53)	1 (5.00)	1 (11.11)	0 (0.00)	8 (4.32)
Carinthia	3 (4.76)	5 (10.42)	1 (2.63)	1 (5.00)	0 (0.00)	0 (0.00)	10 (5.41)
Lower Austria	15 (23.81)	8 (16.67)	5 (13.16)	1 (5.00)	1 (11.11)	2 (28.57)	32 (17.30)
Upper Austria	5 (7.94)	3 (6.25)	1 (2.63)	4 (20.00)	1 (11.11)	1 (14.29)	15 (8.11)
Salzburg	2 (3.17)	0 (0.00)	3 (7.89)	1 (5.00)	0 (0.00)	1 (14.29)	7 (3.78)
Styria	4 (6.35)	3 (6.25)	2 (5.26)	4 (20.00)	1 (11.11)	0 (0.00)	14 (7.57)
Tyrol	4 (6.35)	3 (6.25)	0 (0.00)	1 (5.00)	2 (22.22)	0 (0.00)	10 (5.41)
Vorarlberg	1 (1.59)	1 (2.08)	2 (5.26)	0 (0.00)	0 (0.00)	0 (0.00)	4 (2.16)
Vienna	28 (44.44)	20 (41.67)	18 (47.37)	5 (25.00)	2 (22.22)	2 (28.57)	75 (40.54)
Other	0 (0.00)	4 (8.33)	2 (5.26)	2 (10.00)	1 (11.11)	1 (14.29)	10 (5.41)
Population density, n (%)							
Densely populated	38 (60.32)	26 (54.17)	27 (71.05)	9 (45.00)	5 (55.56)	2 (28.57)	107 (57.84)
Moderately populated	20 (31.75)	15 (31.25)	10 (26.32)	9 (45.00)	3 (33.33)	4 (57.14)	61 (32.97)
Sparsely populated	5 (7.94)	7 (14.58)	1 (2.63)	2 (10.00)	1 (11.11)	1 (14.29)	17 (9.19)
Perceived patient access (based on health profession), n (%)							
Poor	0 (0.00)	7 (14.58)	1 (2.63)	0 (0.00)	2 (22.22)	2 (28.57)	12 (6.49)
Moderate	16 (25.40)	21 (43.75)	13 (34.21)	6 (30.00)	4 (44.44)	2 (28.57)	62 (33.51)
Good	47 (74.60)	20 (41.67)	24 (63.16)	14 (70.00)	3 (33.33)	3 (42.86)	111 (60.00)
Telehealth experience, n (%)							
Never	46 (73.02)	32 (66.67)	31 (81.58)	16 (80.00)	2 (22.22)	4 (57.14)	131 (70.81)
In the past	11 (17.46)	3 (6.25)	2 (5.26)	2 (10.00)	1 (11.11)	1 (14.29)	20 (10.81)
Currently	6 (9.52)	13 (27.08)	5 (13.16)	2 (10.00)	6 (66.67)	2 (28.57)	34 (18.38)

a PT: physiotherapists.

b OT: occupational therapists.

c ORT: orthoptists.

d NUR: nurses.

e SLT: speech and language therapists.

### Telehealth Experience

In total, 71% (131/185) of the participants stated that they had never performed telehealth before, 11% (20/185) had telehealth experience but did not use it currently and 18% (34/185) currently use telehealth.

**Figure 3.**
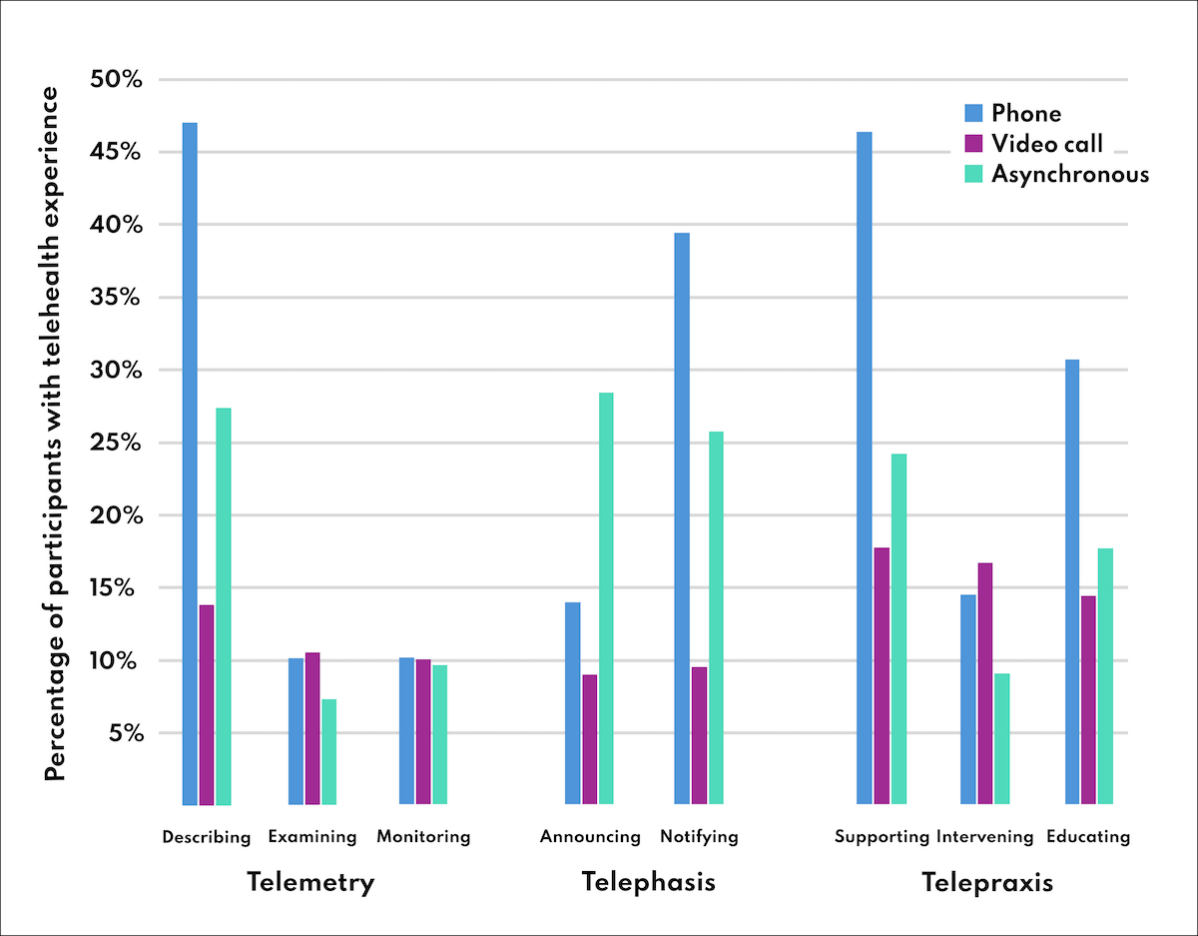
Telehealth experience of the participants. Participants could select all functions they had used; thus, percentages exceed or fall short of 100%.

The participants had used phone or video calls or asynchronous communication for a variety of telehealth services. Telemetry was applied for describing by 95/185 (51%), for examining by 35/185 (19%), and for monitoring by 38/185 (21%); telephasis for announcing by 64/185 (35%), and for notifying by 91/185 (49%); telepraxis for supporting by 100/185 (54%), for intervening by 49/185 (26%), and for educating by 69/185 (37%). Further details on the usage of phone, video call, or asynchronous communication are listed in Figure 3.

### Telehealth Acceptance Pre and Post Web-Based Training

**Figure 4.**
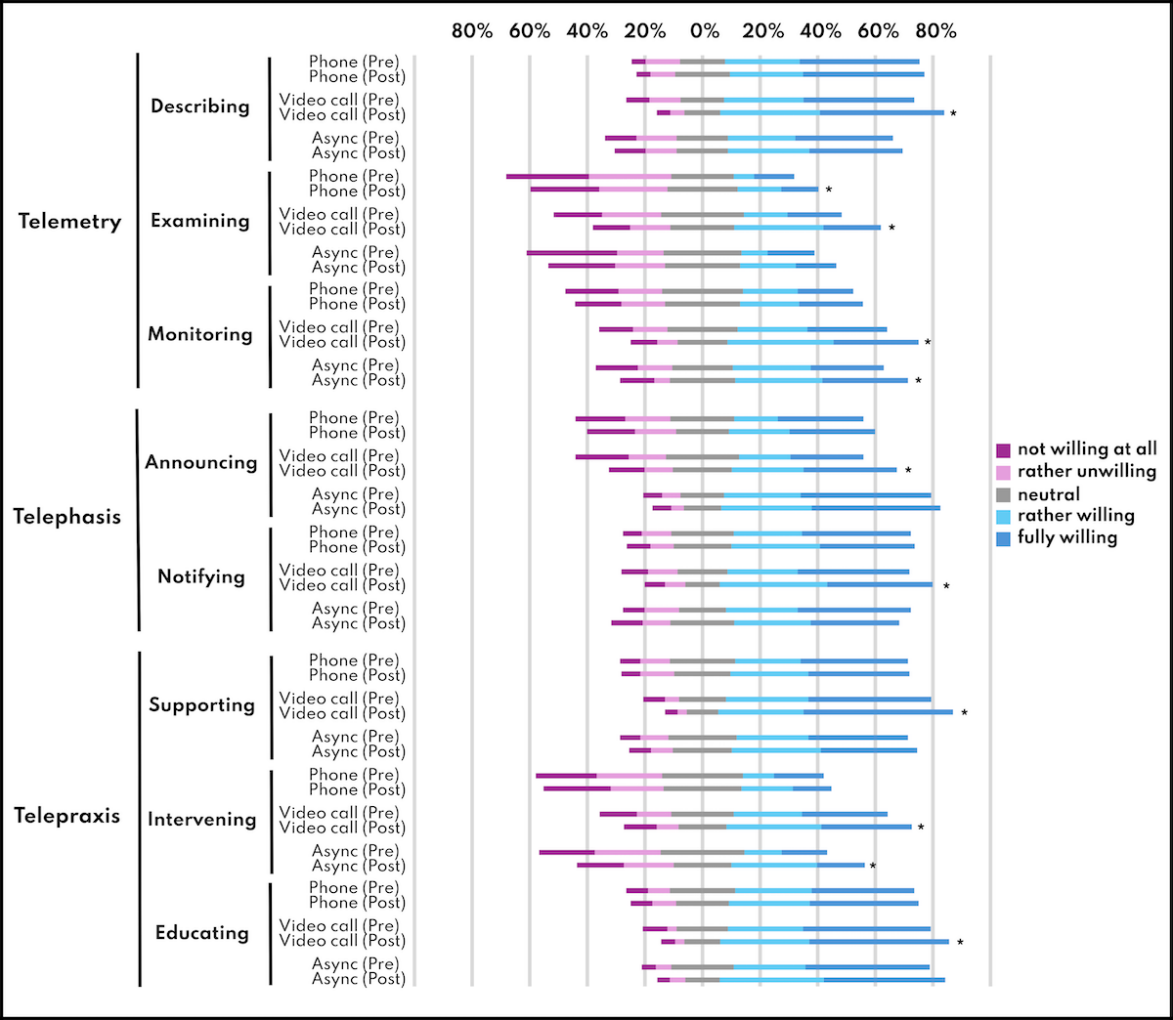
Telehealth acceptance change pre- and post-intervention. The asterisks indicate significant changes.

The total telehealth acceptance (*P*<.001), acceptance for telemetry (*P*<.001) and telepraxis (*P*<.001), acceptance for telehealth via video call (*P*<.001) and asynchronous telehealth (*P*=.007) changed significantly, while acceptance for telephasis (*P*=.203) and acceptance for phone call–based telehealth (*P*=.1) had no significant changes (Table 3 and Figure 4).

**Table 3.** Telehealth acceptance change pre- and post-intervention for telehealth functions, applications, and mediums (n=185).

Function and application	Pre-median acceptance (IQR)	Post-median acceptance (IQR)	*P* value	*r*	Int Hattie^b^
Acceptance total	61 (27)	63 (28)	<.001^a^	0.21	+++
Acceptance telemetry total	20 (13)	23 (12)	<.001^a^	0.20	+++
Describing					
Phone	3 (2)	3 (2)	.429	0.04	N/A
Videocall	3 (2)	3 (1)	<.001^a^	0.20	+++
Asynchronous	3 (3)	3 (2)	.584	0.03	N/A
Examining					
Phone	1 (2)	2 (2)	.018^a^	0.12	++
Videocall	2 (2)	3 (2)	<.001^a^	0.18	++
Asynchronous	2 (3)	2 (2)	.056	0.10	N/A
Monitoring					
Phone	2 (2)	2 (2)	.211	0.06	N/A
Videocall	3 (2)	3 (2)	.003^a^	0.16	++
Asynchronous	3 (3)	3 (2)	.022^a^	0.12	++
Acceptance telephasis total	16 (8)	17 (8)	.203	0.07	N/A
Announcing					
Phone	2 (3)	2 (3)	.352	0.05	N/A
Videocall	2 (3)	3 (2)	<.001^a^	0.20	+++
Asynchronous	3 (2)	3 (1)	.438	0.04	N/A
Notifying					
Phone	3 (2)	3 (2)	.785	0.01	N/A
Videocall	3 (2)	3 (2)	.042^a^	0.11	++
Asynchronous	3 (2)	3 (2)	.051	0.10	N/A
Acceptance telepraxis total	24 (11)	26 (11)	.003^a^	0.16	*++*
Supporting					
Phone	3 (2)	3 (2)	.885	0.01	N/A
Videocall	3 (2)	4 (1)	<.001^a^	0.18	++
Asynchronous	3 (2)	3 (2)	.487	0.04	N/A
Intervening					
Phone	2 (2)	2 (2)	.949	0.00	N/A
Videocall	3 (3)	3 (2)	.029^a^	0.11	++
Asynchronous	2 (2)	2 (2)	.001^a^	0.17	++
Educating					
Phone	3 (2)	3 (2)	.418	0.04	N/A
Videocall	3 (2)	3 (1)	.011^a^	0.13	++
Asynchronous	3 (2)	3 (1)	.123	0.08	N/A
Acceptance phone total	19 (11)	20 (11)	.053	0.10	N/A
Acceptance video call total	22 (10.5)	24 (9.5)	<.001^a^	0.26	*+++*
Acceptance asynchronous total	21 (11)	22 (10)	.007^a^	0.14	*++*

a Significant value, with *P *<.05.

b Effect sizes were calculated using the r statistic, calculated as z/sqrt(n), and interpreted according to Hattie’s guidelines [16,17]. +=developmental effect (no effect). ++=teacher effect (small effect). +++=zone of desired effects (medium effect). Effect interpretation only for significant results.

c N/A: not applicable.

### Influence of Telehealth Barriers Pre and Post Web-Based Training

**Figure 5.**
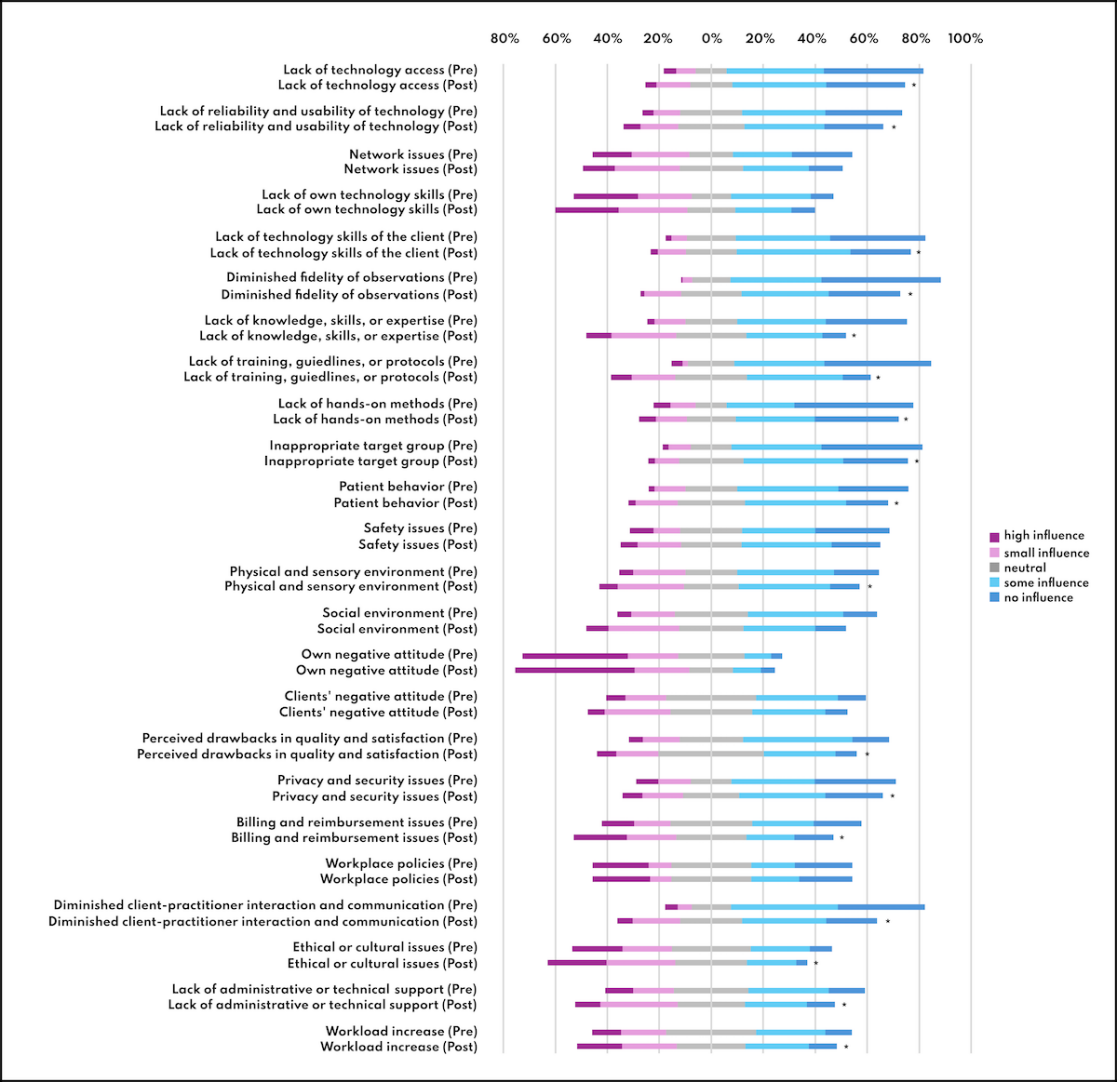
Perceived influence of certain barriers on the willingness to use telehealth pre and post intervention. Asterisk indicates significant changes.

The perceived influence of barriers on the willingness to adopt telehealth also was significantly reduced after the intervention (*P*<.001) (Table 4 and Figure 5).

**Table 4.** Change in the perceived influence of certain barriers on the willingness to adopt telehealth pre- and post-intervention.

Barrier	Pre-MDN (IQR)	Post-MDN (IQR)	*P* value	*r*	Int Hattie^b^
Barriers total	61 (18)	53 (20.5)	<.001	0.39	+++
Lack of technology access	3 (1)	3 (2)	.006^a^	0.14	++
Lack of reliability and usability of technology	3 (2)	3 (1)	.003^a^	0.16	++
Network issues	2 (2)	2 (2)	.055	0.10	not applicable
Lack of own technology skills	2 (3)	1 (2)	.077	0.09	not applicable
Lack of technology skills of the client	3 (2)	3 (1)	<.001^a^	0.20	+++
Diminished fidelity of observations	3 (1)	3 (2)	<.001^a^	0.28	+++
Lack of knowledge, skills, or experience	3 (2)	2 (2)	<.001^a^	0.41	+++
Lack of training, guidelines, or protocols	3 (1)	2 (2)	<.001^a^	0.42	+++
Lack of hands-on methods	3 (2)	3 (2)	.002^a^	0.16	++
Inappropriate target group	3 (2)	3 (2)	.002^a^	0.16	++
Patient behavior	3 (2)	3 (1)	<.001^a^	0.19	++
Safety issues	3 (2)	3 (1)	.059	0.10	not applicable
Physical and sensory environment	3 (2)	2 (2)	.012^a^	0.13	++
Social environment	2 (1)	2 (2)	.294	0.16	not applicable
Own negative attitudes	1 (2)	1 (2)	.294	0.05	not applicable
Clients’ negative attitude	2 (1)	2 (2)	.1	0.09	not applicable
Perceived drawbacks (quality and satisfaction)	3 (1)	2 (1)	<.001^a^	0.20	+++
Privacy and security issues	3 (2)	3 (1)	.042^a^	0.11	++
Billing and reimbursement issues	2 (2)	2 (2)	<.001^a^	0.17	++
Workplace Policies	2 (2)	2 (2)	.815	0.01	not applicable
Diminished client-practitioner interaction and communication	3 (2)	3 (1)	<.001^a^	0.30	+++
Ethical and cultural issues	2 (2)	2 (1)	.002^a^	0.16	++
Lack of administrative and technical support	2 (2)	2 (2)	.003^a^	0.16	++
Workload increase	2 (2)	2 (2)	.032^a^	0.11	++

a Significant value, with *P *<.05.

b Effect sizes were calculated using the r statistic, calculated as z/sqrt (n), and interpreted according to Hattie’s guidelines [16,17]. +=developmental effect (no effect). ++=teacher effect (small effect). +++=zone of desired effects (medium effect). Effect interpretation only for significant results.

### Satisfaction With Web-Based Training

Overall satisfaction ratings were relatively high, with a total median of 76 (IQR 13) across all participants (Table 5).

**Table 5.** Median satisfaction scores (with IQR) of the Training Evaluation Inventory (TEI) for the web-based training.

Satisfaction	SLT^a^ (n=9)	OT^b^ (n=48)	PT^c^ (n=63)	ORT^d^ (n=38)	NUR^e^ (n=20)	Other (n=7)	Total (n=185)
Total satisfaction	83 (7)	76 (13.75)	76 (11)	73 (14.25)	71.5 (18.5)	74 (28)	76 (13)
Subjective enjoyment							
Overall, I liked the course.	4 (1)	3 (1)	3 (1)	3 (1)	3 (1)	4 (1)	3.29
The learning atmosphere was agreeable.	4 (0)	4 (1)	4 (1)	4 (1)	3 (1)	4 (1)	3.43
The learning was fun.	4 (1)	3 (2)	3 (2)	3 (2)	3 (2)	3 (3)	2.88
Perceived usefulness							
I find the course useful for my job.	4 (1)	3 (1)	4 (1)	3 (2)	4 (1)	3 (1)	3.24
Investing time in this course was useful.	4 (1)	3 (1)	3 (1)	3.5 (1)	3 (1)	3 (1)	3.33
I can apply the content of this course in my job.	3 (1)	3 (1)	3 (1)	2 (1)	2.5 (1)	3 (1)	2.59
I derive personal use from this course.	3 (1)	3 (1)	3 (1)	3 (2)	3 (2)	3 (2)	2.98
Perceived difficulty							
The contents were comprehensible.	4 (0)	4 (1)	4 (0)	4 (0)	4 (1)	4 (0)	3.69
The language (foreign words and technical terms) was comprehensible.	4 (0)	4 (0)	4 (0)	4 (0)	4 (0)	4 (0)	3.77
I kept up thematically in the course.	4 (0)	4 (0)	4 (0)	4 (0)	4 (1)	4 (0)	3.76
The time was sufficient for the themes covered.	4 (0)	4 (1)	4 (0)	4 (0)	4 (1)	4 (2)	3.65
Subjective knowledge gain							
I have the impression that my knowledge has expanded on a long-term basis.	3 (1)	3 (1)	3 (1)	3 (1)	3 (1)	3 (2)	3.09
I will be able to remember the new themes well.	3 (1)	3 (1)	3 (2)	3 (2)	3 (1)	3 (1)	2.94
I think that I will still be able to report what I learned some time after the course.	3 (1)	3 (2)	3 (1)	3 (0)	3 (1)	3 (2)	2.92
Attitude towards training							
I will apply what I learned to my day-to-day work.	3 (2)	2 (1)	2 (1)	2 (2)	2.5 (3)	2 (3)	2.24
I find it good that basics of telehealth were imparted and discussed.	4 (0)	4 (0)	4 (0)	4 (1)	4 (1)	4 (2)	3.7
I find it good that forms of application of telehealth were imparted and discussed.	4 (0)	4 (0)	4 (0)	4 (1)	4 (1)	4 (1)	3.72
I find it good that legal aspects of telehealth were imparted and discussed.	4 (0)	4 (0)	4 (0)	4 (1)	4 (1)	4 (1)	3.67
I find it good that technical aspects of telehealth were imparted and discussed.	4 (0)	4 (1)	4 (1)	4 (1)	4 (1)	4 (3)	3.54
I find it good that practical applications of telehealth were imparted and discussed.	4 (0)	4 (0)	4 (0)	4 (1)	4 (1)	4 (1)	3.69
I find it good that profession-specific aspects of telehealth were imparted and discussed.	4 (0)	4 (0)	4 (0)	4 (0)	4 (1)	4 (4)	3.78
I would recommend this course to my colleagues.	4 (1)	4 (1)	3 (1)	3 (1)	4 (1)	3 (4)	3.35

a SLT: speech and language therapists.

b OT: occupational therapists.

c PT: physiotherapists.

d ORT: orthoptists.

e NUR: nurses.

### Qualitative Results

Participants across all feedback sources generally found the telehealth training informative, well-structured, and valuable. They appreciated the comprehensive overview of telehealth, the professional presentation style, and the flexibility offered by the web-based format. The training expanded their understanding of telehealth possibilities, especially for those with little to no prior experience. However, participants expressed a desire for more practical examples, interactive elements, and ongoing support to bridge the gap between theory and practice. Concerns were raised about challenges in implementing telehealth, particularly regarding data protection, patient accessibility, and institutional barriers. More details on the qualitative results can be found in Multimedia Appendix 2.

## Discussion

### Principal Findings

This study, to our knowledge, is the first to evaluate the effect of an interprofessional on-demand postgraduate telehealth course for health care professionals on acceptance of telehealth and their perceived barriers to its adoption. The main findings indicate a significant positive impact, underscoring the value of structured telehealth education. According to the Technology Acceptance Model, the intention to use a technology is primarily driven by 2 key factors: perceived usefulness and perceived ease of use [19]. Our results suggest the course might affect those key factors, but its impact on telehealth use behavior remains unclear.

### Key Findings and Their Implications

The results showed a significant increase in telehealth acceptance and a significant reduction in perceived barriers among participants, consistent with previous research that highlights the role of training in improving telehealth knowledge and confidence [20,21].

Participants acknowledged the informative and structured nature of the course, aligning with studies that emphasize the importance of tailored web-based learning for health care providers [22]. This highlights the critical need for comprehensive telehealth training, particularly for practitioners with little to no prior telehealth experience, who represented the majority in this study (71%).

Participants identified key challenges, including concerns about data protection, patient accessibility, and institutional barriers, echoing previous findings in telehealth literature [2,3,23]. Addressing these issues through additional practical examples, interactive learning opportunities, and ongoing support could further enhance the effectiveness of telehealth training.

The most significant result was the increase in overall telehealth acceptance after the intervention. Particularly, the acceptance of telemetry and telepraxis increased, as well as the acceptance of video call-based telehealth and of asynchronous telehealth. This suggests that the training helped them to envision a broader range of applications and modalities for delivering remote care. While improvements were noted in several domains, some—such as telephasis and phone-based telehealth—did not show significant changes. The stability in these areas may reflect that certain forms of telehealth were already well understood or already integrated into the workflows, such as, eg, notifying patients by phone.

Additionally, it is important to consider that while the training significantly increased participants’ intention to adopt telehealth, this does not necessarily translate into its actual use in daily practice. Incorporating more hands-on training components by establishing blended-learning formats with practical simulations or real-time demonstrations could facilitate the transition from intention to actual use.

Beyond simply making professionals more open to telehealth, the training also reduced the perceived influence of a wide range of barriers. Before the intervention, participants identified multiple obstacles. After completing the course, there were notable reductions in perceived barriers. This finding indicates that structured education can address knowledge deficits and clarify implementation pathways, thereby making telehealth seem more feasible. The course appeared most effective in reducing concerns related to a lack of training and standardized guidelines. By providing clear information on legal frameworks, technology usage, and practical strategies, the training directly addressed these issues. Barriers linked to patient factors and the therapeutic environment were also diminished. Notably, participants’ perceived drawbacks regarding telehealth quality and satisfaction, as well as concerns about reduced client-practitioner interaction and communication, were lowered following the training. One possible reason for this improvement is that the course provided concrete strategies and examples of how telehealth can maintain, or even enhance, meaningful therapeutic engagement. However, some barriers were not significantly reduced by the web-based course, such as individual technology skills, network, or safety issues. The course also did not influence either the barrier of clients’ or the practitioners’ negative attitudes toward telehealth. Maybe lowering those barriers requires more focus on experiential learning, systemic change, or a more sustained intervention.

The qualitative findings underscore the value participants placed on the course’s comprehensive, well-structured content and profession-specific modules. At the same time, they highlight a need for ongoing, practical, and interactive support to embed telehealth into everyday practice. Addressing unresolved questions—such as establishing mentorship channels, refining legal guidance, and developing strategies for patient engagement—will be critical to ensure that telehealth initiatives are both clinically viable and inclusive.

These insights serve as a roadmap for future iterations of the training. Given the significant attitude shifts observed after only 2 hours of content, such short “gateway” modules could be deployed first to boost health professionals’ readiness before rolling out more intensive skill-based or certification courses. Incorporating blended learning and flipped classroom settings, more hands-on examples, offering supplementary materials, and facilitating interactive forums could further amplify the course’s impact. By combining either self-developed or third-party massive open web-based courses or open educational resources content with synchronous, hands-on sessions, educators could create a rich, interactive learning environment. Learners can engage with the theoretical components digitally at their own pace, while live sessions focus on practical exercises, discussions, and application of telehealth strategies. By integrating these educational strategies, future training initiatives can better equip health care professionals with both the conceptual understanding and practical skills necessary to overcome barriers to telehealth adoption.

For policymakers, the results highlight the importance of fostering a supportive environment that translates EU-level digital health policies into effective national education strategies. Policy makers should collaborate with academic institutions to promote the adoption of standardized telehealth training programs and to integrate digital competencies into health education.

### Strengths and Contributions

The study provides valuable insights into telehealth education, particularly its ability to enhance acceptance and reduce barriers across diverse professional groups. The inclusion of a broad participant base from various health care disciplines reflects the cross-sectoral applicability of telehealth education, aligning with prior research that underscores the interdisciplinary relevance of telehealth [5,24].

Moreover, the findings support the notion that telehealth training fosters not only technological acceptance but also a broader understanding of telehealth’s potential applications. This is particularly relevant considering the resources of specialist staff and increasing demand for digital skills and remote therapy and care delivery, as identified in earlier studies [25,26].

### Limitations

Despite its strengths, the study has several limitations that warrant consideration. Due to the design of the study, a control group was absent. Without a control group, it is difficult to attribute observed changes solely to the intervention. Future studies should consider randomized controlled designs to strengthen causal inferences.

Another limitation is the representativeness of the study population. The participants were self-selected, which may limit the generalizability of the findings to the broader population of health care professionals. Also, because professional associations deployed the participation link via differing formats (website postings, newsletters, and direct emails), we cannot determine the exact number of recipients or calculate a precise response rate, which may affect assessments of self-selection bias.

The third limitation corresponds with the uncertainty of course content exposure due to the on-demand character of the training. The web-based format did not guarantee that participants fully engaged with the course content, potentially impacting the results. This limitation was mitigated by filtering the results and excluding participants who did not engage with at least 40 minutes of the total 120-minute video content in the analysis. This threshold was established to ensure sufficient exposure to the intervention.

### Future Directions

Building on these findings, future research should explore the following main aspects to broaden the findings. A future study should also examine long-term effects of telehealth education on clinical practice and patient outcomes, including a follow-up assessment. In addition, education programs would benefit from practical training enhancements, such as case-based simulations and interactive modules to bridge the gap between theory and application.

Finally, integrating telehealth education into initial health care training programs could ensure that future professionals are better prepared to embrace telehealth as part of routine care delivery. To achieve policy goals, significant investments in digital infrastructure and targeted workforce training are needed to turn technological potential into practical use. Building partnerships across sectors and creating ongoing feedback among regulators, health care organizations, and practitioners can help ensure that telehealth initiatives remain flexible, sustainable, and responsive to the evolving needs of clinical practice and patients.

### Conclusions

This study reinforces the critical role of structured telehealth education in enhancing health care professionals’ acceptance of telehealth and reducing perceived barriers. While challenges remain, particularly in implementation and institutional support, the findings may indicate the potential of short-term, remote, and targeted training programs to advance telehealth adoption. We recommend systematically integrating telehealth modules into continuing professional development and national licensing requirements. Further research and investment in education and infrastructure are essential to fully realize the benefits of telehealth and digitalization in modern health care.

## Supplementary material

10.2196/74107 Multimedia Appendix 1 Course details.

10.2196/74107 Multimedia Appendix 2 Qualitative results.
