# Supplementary material for: Telehealth Acceptance and Perceived Barriers Among Health Professionals: Pre-Post Evaluation of a Web-Based Telehealth Course
Source: JMIR Hum Factors. 2025 Sep 3;12:e74107. doi: 10.2196/74107 (PMC12408057; doi:10.2196/74107)
Supplement: Multimedia Appendix 1 [file humanfactors-v12-e74107-s001.pdf]

GENERAL PART

Part 1 – Concept clarification

1. **Traditional Healthcare**: Involves physical visits to healthcare providers, overcoming the geographic distance through travel, which takes time and incurs costs.
2. **Telehealth**: Uses Information and Communication Technologies (ICT) to bridge the physical distance between healthcare providers and patients. Examples include video consultations, phone calls, or data exchanges (such as sending medical reports via email or apps).
3. **Definitions and Context**: Telehealth is a subset of the broader "Digital Health" or "eHealth" field, which involves using electronic tools to improve healthcare. Telehealth specifically focuses on using ICT to provide healthcare services over long distances, encompassing clinical care, education, and administration.
4. **Telemedicine**: A more specific subset of telehealth, which refers exclusively to medical diagnostic and treatment services delivered remotely using ICT.
5. **Other Related Terms**:
    - **mHealth**: Mobile health, involving mobile devices like phones or tablets for healthcare purposes.
    - **Telerehabilitation**: Remote rehabilitation services.
    - **Teletherapy**: Remote therapeutic interventions.
    - **Telecare**: Remote nursing or caregiving services.

The presentation highlights how these terms often overlap and are not always clearly defined but aim to deliver healthcare more efficiently and cost-effectively.

Part 2 – Application forms

1. **Synchronous Telehealth**:
    - Involves real-time communication between patients and healthcare providers, which strengthens the therapeutic alliance.
    - Advantages: Immediate interaction.
    - Disadvantages: Limited to scheduled times, hands-on procedures are not possible.
2. **Asynchronous Telehealth**:
    - Involves time-delayed communication (e.g., messaging, pre-recorded videos).
    - Advantages: Flexible and not dependent on both parties being available simultaneously.
    - Disadvantages: No immediate interaction and limited physical assessment.
3. **Telemonitoring**:
    - Remote tracking of health metrics like blood sugar or heart rate.
    - Advantages: Continuous data collection for better diagnosis and prevention.

    - Disadvantages: Compliance and data privacy concerns, indirect patient-provider contact.
4. **mHealth (Mobile Health)**:
    - Mobile applications on devices like smartphones and smartwatches.
    - Examples: Apps for preventive care, education, or medical monitoring.
    - In some countries (e.g., Germany), digital health applications (DiGA) are reimbursable by health insurance, but legal issues remain in other places like Austria.
5. **Blended Care/Hybrid Models**:
    - Combination of in-person and telehealth services, offering flexibility in treatment.
    - Hybrid models might include both in-person and remote participants simultaneously, such as in group therapy sessions.
6. **Telehealth for Public Health**:
    - Facilitates communication between healthcare providers and patients, but also among healthcare professionals for training, conferences, and public health efforts.

The presentation provides an in-depth exploration of how telehealth can be applied across different domains, addressing the potential benefits and challenges for each method.

Part 3 – Legal Aspects

1. **MTD Law (2024)**:
    - Governs medical-therapeutic-diagnostic health professions.
    - Allows telehealth if professional standards are met and patients are informed of the unique aspects of telehealth.
2. **GuK Law**:
    - Regulates health and nursing professions.
    - Telehealth is permissible but must align with direct and personal care mandates.
3. **Data Protection and Privacy**
    - **GDPR (General Data Protection Regulation)**:
        - Emphasizes data protection principles such as legality, purpose limitation, data minimization, and accountability.
        - Special conditions for processing sensitive health data.
    - **Health Telematics Law (2012):**
        - Establishes security standards for processing electronic health data, including genetic information.
    - **Medical Device Regulation (2021):**
        - Outlines requirements for medical software and devices used in telehealth.
4. **Practical Considerations for Telehealth**
    - Ensuring software complies with GDPR and, if necessary, qualifies as a medical product.
    - Implementing robust data security measures: Password protection, data backups, and encrypted communication; Avoiding free or unsecured cloud services for sensitive data

handling; Using tools like TOSDR and MARS to evaluate software privacy and therapeutic quality.

5. **Organizational Aspects**
   - Drafting treatment contracts and terms of service (AGBs).
   - Obtaining informed consent for telehealth usage.
   - Clarifying insurance coverage and reimbursement processes.
   - Referencing agreements like Austria's ÖGK framework for telehealth reimbursement.
6. **Recommendations**
   - Regularly consult with professional associations for updates on legal and practical telehealth aspects.
   - Incorporate structured guidelines to align telehealth practices with existing laws and ethical standards.
7. **Concluding Notes**
   - Legal considerations are critical for implementing telehealth responsibly and effectively.
   - Adherence to national and international regulations ensures patient safety, privacy, and service quality.

Part 4 – Technology Aspects

1. **Technical Requirements for Telehealth**:
   - Importance of reliable hardware (PC, smartphones, tablets), software, and internet connectivity.
   - Security features such as encryption (end-to-end) and GDPR compliance are critical, especially when handling sensitive health data.
2. **Digital Trends in Home-Based Rehabilitation**:
   - Technologies include sensors, robotics, gamification, virtual and augmented reality, mobile apps, and digital platforms. These help with remote rehabilitation and patient engagement.
3. **Communication Methods in Telehealth**:
   - Various communication tools (phone calls, emails, video conferencing, messaging services) are used, each with specific advantages and limitations in terms of privacy, immediacy, and data security.
4. **Technical Barriers and Facilitators**:
   - Common barriers include limited access to technology, poor network quality, and lack of technical skills among patients and providers.
   - Facilitators involve using simple, user-friendly technology, offering step-by-step guides, and providing adequate training to both patients and providers.
5. **Pros and Cons of Different Technologies**:
   - **Synchronous Methods** (e.g., video calls, phone calls): Immediate communication and visual inspection are possible but require a stable internet connection and appropriate hardware.
   - **Asynchronous Methods** (e.g., email, messaging, apps): More flexible but lack real-time interaction and can raise privacy concerns.
6. **Data Security Concerns**:

- The use of unsecured communication methods (e.g., email, WhatsApp) is generally discouraged due to privacy and compliance issues. Proper encryption and data protection protocols are recommended.

7. **Examples of mHealth Applications**:
   - The presentation mentions DiGA (Digital Health Applications) in Germany, which are reimbursable by insurance and can be prescribed by doctors. These apps aid in diagnosis, therapy, or health monitoring.

Part 5 – Practical implementation

1. **Common Barriers to Practical Implementation**:
   - Limited ability to use hands for treatment.
   - Reduced capacity for observation and interpretation.
   - Communication and interaction constraints.
   - Lack of knowledge, experience, and protocols related to telehealth.
   - Unsuitability of certain client groups or environments.
   - Concerns about safety and negative perceptions regarding the quality of telehealth.
2. **Key Requirements for Telehealth**:
   - **Need**: Factors like mobility issues, travel difficulties, availability of healthcare providers, and the desire for more self-management can drive the demand for telehealth.
   - **Suitability**: Success in telehealth depends on acceptance by both patients and providers, as well as compliance with treatment plans, security, and appropriate technology use.
3. **Acceptance and Understanding**:
   - The provider's acceptance of telehealth is crucial for patient buy-in.
   - Demonstrating the benefits of telehealth and introducing blended-care models can improve acceptance.
4. **Managing Adherence and Compliance**:
   - Focus is needed on treatment quality, data accuracy, ethics, and patient safety, especially with asynchronous telehealth.
5. **Safety Considerations**:
   - Ensuring safety involves preventing fall or injury risks, selecting appropriate exercises, and involving competent caregivers when necessary.
6. **Adapting Treatment Methods**:
   - Practitioners must consider how to modify traditional treatment techniques for a virtual setting, enhancing video quality and guiding patients through self-management and education.
7. **Maintaining Patient Relationships**:
   - Relationship-building is essential, whether through initial in-person meetings or using techniques like eye contact, gestures, and playful elements during video calls.
8. **Spatial and Equipment Considerations**:
   - The telehealth environment should ensure adequate space, necessary materials, and privacy for both the provider and patient. Equipment like wireless headsets and high-quality cameras/microphones can improve the experience.
9. **Handling Sensory, Cognitive, and Motor Impairments**:

- Telehealth should be adapted for patients with disabilities, using accessible technology (e.g., screen readers, Braille displays), high-contrast visuals, and involving support persons where needed.

PROFESSION SPECIFIC PART

Occupational therapy

1. **Telehealth Applications in Occupational Therapy**:
   - Includes both synchronous (real-time) and asynchronous (delayed) methods, as well as mHealth (mobile health) standalone applications.
   - Examples include:
     - **CO-OP via Telehealth**: A cognitive approach for various tasks (e.g., daily activities, work, school) used in neurology, pediatrics, and psychiatry.
     - **Hand Therapy via Telehealth**: Remote treatment for hand injuries or conditions with initial in-person assessments followed by telehealth sessions.
2. **Synchronous Applications**:
   - **CO-OP (Cognitive Orientation to daily Occupational Performance)**:
     - A cognitive strategy intervention with evidence supporting its effectiveness, though it's not widespread, and there are limited training opportunities.
   - **Hand Therapy**:
     - Telehealth is found to be useful in monitoring hand conditions and tracking progress, though it lacks the hands-on feedback available in-person.
3. **Asynchronous Applications**:
   - **ReHand**: A mobile app for hand, wrist, and finger rehabilitation, offering evidence-based exercise plans that can be tailored to the patient's needs.
   - **Therap.io**: An app providing personalized training plans, pain tracking, and exercise creation with free access. However, it lacks integration with practice management software.
   - **Owaves**: A time structuring app used for daily planning with visual features and reminders to help maintain occupational balance.
   - **MapIt**: A tool for remote home environment assessments, though it has limitations in user-friendliness and availability in multiple languages.
   - **MyReha**: A cognitive training app (including aphasia training) developed in Austria, allowing therapists to monitor and adjust treatments remotely.
4. **mHealth (Mobile Health) Standalone Applications**:
   - **elsa ADHS**: A mobile app providing tips for parents of children with ADHD, offering practical advice for daily life in various settings (home, school).
   - **Daylio**: A diary app for tracking mood and activities, with features like password protection and export capabilities.

Speech and language therapy

1. **Research Background**

- Studies confirm the effectiveness of telehealth interventions for various speech disorders, including dysphonia, stuttering, aphasia, dysphagia, and speech development disorders.
- Emphasis on tailored formats based on specific disorders.
- Building therapeutic relationships is possible but requires active effort in telehealth settings.

2. **Applications in Logopedics**
   - **Synchronous Tools**:
     - Software like Appointmed for video conferencing, patient management, and documentation.
     - Integration with tools like Whereby and Miro for interactive sessions.
   - **Asynchronous Tools**:
     - Apps such as MyReha, SpeechCare, LingoTalk, and PhonoLo for exercises in aphasia therapy, cognitive training, and articulation.
   - **Standalone Apps**:
     - Neolexon: Apps for articulation training in children and aphasia rehabilitation in adults, including interactive modules and customizable features.
     - Other apps like Tactus, Besser Sprechen, and Delayed Auditory Feedback (DAF) cater to specific therapy needs.
3. **Technological Aspects**
   - Tools like Miro for interactive whiteboarding.
   - Importance of setup in video conferences (lighting, secondary cameras, quality audio devices).
4. **Challenges and Limitations**
   - Costs and lack of insurance coverage for some tools.
   - Issues with speech recognition in dialects or noisy environments.
   - Limited therapist contact in standalone app models.
5. **Future Developments**
   - Projects like DysarTrain (dysarthria training) and Dys-Phappy (therapy for older dysphagia patients) are in progress.
   - Aim to address gaps for immobile or hard-to-reach patients.
6. **Quality Criteria for Telehealth Tools**
   - Evidence-based design and transparent cost structures.
   - Compatibility with professional expertise.
   - Accessible support and realistic therapeutic claims.
7. **Concluding Notes**
   - Telehealth enhances therapy frequency and transparency in logopedics.
   - Helps overcome mobility and distance barriers.
   - Boosts patient motivation and practice regularity.
   - Intended as a complementary approach, not a replacement for traditional methods.

Physiotherapy

1. **Overview of Telehealth in Physiotherapy**
   - General information, including FAQs about regulations, reimbursement, and suitability for specific patient groups.

- Examples of synchronous, asynchronous, and mHealth applications for physiotherapy.

2. **FAQs**
   a. **International Telehealth Practice**:
      - Usually requires recognition of qualifications (nostrification) and registration in the relevant country's healthcare professional registry.

   b. **Insurance Coverage**:
      - Teletherapy is reimbursable in Austria under agreements like §18 and §15 of the ÖGK framework.

   c. **Legal Requirements**:
      - Consent for telehealth must be signed by legal representatives for minors.

   d. **Online Group Therapy**:
      - Feasible under similar regulations as in-person sessions, with specific software limitations (e.g., mostly 1-to-1 connections).

3. **Scientific Evidence on App Quality**
   - Study by Paganini et al. (2021) analyzed 312 apps using the Mobile Application Rating Scale (MARS).
   - Findings:
     - Moderate overall quality (mean score 3.6/5).
     - Significant issues with data security and privacy features.
     - Lack of integrated emergency functionalities.

4. **Examples of Telehealth Applications**
   - **Synchronous Tools**:
     - Visit-e: Free tool for video communication and scheduling, supports open-source frameworks like Jitsi and FHIR standards.
   - **Asynchronous Tools**:
     - Therap.io: Allows user management, personalized exercise plans, and pain tracking; CE-certified and GDPR-compliant.
   - **mHealth Standalone Applications**:
     - **Visible**: For Long-COVID and ME/CFS symptom tracking; includes features like heart rate and HRV monitoring.
     - **Kaia**: A certified digital health app (DiGA) for back pain management, combining exercises, relaxation, and education.

5. **Key Features of mHealth Applications**
   - Common functionalities include symptom tracking, exercise customization, and integration with health metrics (e.g., photoplethysmography for HRV).
   - Challenges include limited integration with therapeutic practices, high costs, and language availability.

6. **Regulatory and Implementation Challenges**
   - Most digital health apps (DiGA) are designed as standalone tools and require physician prescriptions in Germany.
   - Pilot projects in Austria are underway to expand the use of such apps in physiotherapy.

7. **Concluding Notes**
   - Telehealth in physiotherapy offers enhanced accessibility and complements traditional methods.
   - Challenges include technological limitations, regulatory hurdles, and the need for better app quality standards.

Nursing

1. **Definition of Telecare (Telepflege)**
   - Utilizes ICT to deliver and coordinate nursing care over a distance.
   - Integrated into connected healthcare systems, focusing on remote exchange of health data and proactive care.
   - Aims to improve patient and client healthcare.
2. **Scope of Telecare**
   - Communication with patients, caregivers, and professionals.
   - Augments or replaces traditional nursing services.
   - Classified into five categories:
     - Counseling, education, and instruction.
     - Wound management.
     - Technical assistance systems.
     - Robotics.
     - Data exchange and electronic documentation.
3. **Potential Benefits**
   - Improves care access for remote or immobile patients.
   - Encourages interaction and preparation for virtual visits.
   - Enhances inclusion of distant family members.
4. **Applications of Telehealth in Nursing**
   - **Synchronous Tools**:
     - *Alles Clara*: Connects caregivers with advisors via chat; supports psychological and nursing advice.
   - **Asynchronous Tools**:
     - *HerzMobil Tirol*: Manages hypertension with a program that tracks vital parameters and provides remote evaluation.
   - **mHealth Standalone Apps**:
     - *Die WundApp*: Supports wound management by recommending materials and assisting decisions.
     - *Wund-Doku App*: Documents wounds with photos and tracks healing progress; GDPR-compliant.
5. **Evaluation of Tools**
   - Tools like *Alles Clara* and *HerzMobil Tirol* offer intuitive platforms with limitations such as reduced functionality or regional availability.
   - Apps like *Die WundApp* and *Wund-Doku App* provide valuable documentation and monitoring but face challenges in integration with broader IT systems.
6. **Experiences with Telehealth**
   - Testimonials from caregivers and patients indicate:
     - Initial hesitance but later acceptance of virtual care.
     - Enhanced quality of life for immobile clients.
     - Increased engagement and preparation by patients for virtual appointments.
7. **Challenges and Considerations**
   - Telehealth is a valuable complement, not a substitute, for in-person care.

- Requires transparency about medical product certifications.
- Pilot projects are ongoing; services are not yet widely available in Austria.

8. **Concluding Notes**
   - Telecare enhances nursing processes through technology but necessitates well-planned implementation and further development for broader adoption.

Orthoptics

1. **Background**
   - Telehealth evolution from early applications (e.g., NASA's Apollo missions) to current teleorthoptics.
   - Accelerated adoption during COVID-19 due to restrictions.
2. **Applications in Teleorthoptics**
   - **Synchronous Tools**:
     - Tools like *Docsy* and *Mein Arzt Online* provide video consultations, patient management, and compliance with GDPR.
   - **Asynchronous Tools**:
     - *Caterna*: Web-based stimulation therapy for amblyopia.
     - *Amblyoplay*: Interactive and personalized therapy for children.
     - *9 Gaze*: Tool for documenting eye motility and visual functions.
   - **mHealth Standalone Applications**:
     - *Piratoplast*: A playful app to encourage children to use eye patches effectively.
3. **Key Features of Applications**
   - Synchronous tools allow real-time interaction and are customizable.
   - Asynchronous tools emphasize motivation, accessibility, and evidence-based outcomes.
   - Standalone apps cater to home-based therapy with features like gamification and progress tracking.
4. **Advantages and Limitations**
   - Advantages:
     - Enhances motivation and therapy compliance.
     - Provides flexibility and accessibility for patients.
     - Supports remote documentation and monitoring.
   - Limitations:
     - Costs for users and healthcare providers.
     - Dependence on internet and device availability.
     - Uncertainty regarding medical product certification for some tools.
5. **Innovative Uses**
   - VR applications like *Luminopia* and *Olleyes* for screening and therapy.
   - Use of dichoptic masking and contrast reduction for amblyopia therapy.
6. **Insights from Expert Interviews**
   - Telehealth's primary focus in orthoptics:
     - 43% on therapy and rehabilitation.
     - 14% on education.
   - Limitations include technical issues, privacy concerns, and program availability.

- Telehealth is valuable for therapy and consultation but less applicable for detailed diagnostics like refraction or CT scans.

7. **Discussion and Future Outlook**
   - Telehealth is increasingly relevant for orthoptic therapy and patient engagement.
   - Emerging technologies, including AI and VR, have potential for broader application.
   - Collaboration between developers, healthcare professionals, and policymakers is crucial for optimizing these tools.
8. **Conclusion**
   - Telehealth in orthoptics complements traditional methods by enhancing accessibility and patient participation.
   - Future developments aim to address current limitations and expand applicability.
