# Supplementary material for: Telehealth Acceptance and Perceived Barriers Among Health Professionals: Pre-Post Evaluation of a Web-Based Telehealth Course
Source: JMIR Hum Factors. 2025 Sep 3;12:e74107. doi: 10.2196/74107 (PMC12408057; doi:10.2196/74107)
Supplement: Multimedia Appendix 2 [file humanfactors-v12-e74107-s002.pdf]

Qualitative results - Details

Positive Aspects	Suggestions for Improvement	Concerns and Critiques	Unresolved Questions
**Content and Structure:** The course was praised for its clear slides, logical organization, and effective coverage of both general and profession-specific information. Participants appreciated the balance between general and specific content.	**Provision of Handouts and Materials:** Desire for downloadable handouts, slides, or transcripts to accompany the videos, aiding note-taking and allowing for review and reinforcement of the material.	**Overwhelming Information:** Some participants felt inundated by the extensive information, particularly on legal and technical aspects.	**Availability of Support and Guidance:** Are there designated contact persons or resources within professional associations or organizations to assist with telehealth implementation? How can practitioners access ongoing support?
**Comprehensive Coverage:** The training provided a broad and detailed overview of telehealth, introducing various forms and possibilities, which was particularly valuable for newcomers to the topic.	**Ability to Re-watch Videos:** Allow participants to revisit the video content multiple times within a certain timeframe to better understand complex topics.	**Lack of Practical Application:** The training could benefit from more practical examples and demonstrations to bridge the gap between theory and practice.	**Practical Implementation Strategies:** How can practitioners learn from others' experiences to understand best practices and avoid common pitfalls in implementing telehealth?
**Engaging Presentation:** Participants found the presenters' speech clear, with appropriate pacing. The use of multiple speakers kept them attentive and made the material more accessible.	**Inclusion of Practical Examples:** Incorporate practical demonstrations. case studies, or real-life scenarios to make the content more tangible and help participants envision how to implement telehealth in their specific contexts.	**E-Learning Limitations:** Some found e-learning less engaging than in-person sessions, missing the opportunity for immediate discussion and interaction.	**Patient Acceptance and Training:** How can healthcare professionals encourage patient acceptance of telehealth, particularly among those less familiar with technology? Are there strategies or resources to support patients in adopting telehealth?
**Flexibility and Accessibility:** The online, asynchronous format allowed	**Opportunities for Interaction and Discussion:** Include interactive elements	**Patient Accessibility and Acceptance:** Concerns about	**Integration into Education:** Can telehealth training be made available to

participants to engage with the videos at their own pace, fitting it into their schedules. Some valued the ability to pause and resume the course.	such as live sessions, or discussion forums, to enhance engagement. Some participants missed the interactive element of in-person training.	patients, especially older adults or those with technological limitations, being overwhelmed by telehealth technology, potentially leading to exclusion.	students or integrated into educational curricula across institutions to prepare future professionals?
**Increased Awareness and Knowledge:** The training expanded participants' understanding of telehealth, revealing aspects and applications they had not previously considered. It was timely and essential for staying current with digital advancements in healthcare.	**Follow-up Training with Practical Focus:** Interest in subsequent courses focusing on practical application, including input from practitioners who have experience with telehealth, to learn best practices and avoid common pitfalls.	**Implementation Challenges:** Difficulties in applying telehealth within clinical settings due to data protection issues, institutional constraints, and lack of support from organizations. Some lacked support from their organizations.	**Access to Practical Examples and Case Studies:** Where can practitioners find practical examples or case studies demonstrating successful telehealth applications to better understand effective implementation?
**Profession-Specific Information:** Inclusion of content tailored to specific professions (e.g., orthoptists, nurses, speech therapists, physiotherapists) was highlighted as especially beneficial, allowing participants to relate the information directly to their fields.	**Interprofessional Collaboration:** Encourage interprofessional learning by including both interprofessional and profession-specific content to enhance understanding and collaboration across disciplines.	**Absence of Support Structures:** Uncertainty about where to seek assistance or guidance in implementing telehealth, highlighting the need for dedicated support channels within professional associations or institutions.	**Data Protection Solutions:** How can practitioners navigate data protection regulations to implement telehealth solutions effectively while ensuring compliance? Specific recommendations for GDPR-compliant telehealth platforms.
**Useful Tools and Resources:** Participants discovered new apps and tools that could aid in their practice, enhancing readiness to adopt telehealth services.	**Technical Enhancements:** Suggestions included shorter video segments (10-15 minutes), options to adjust playback speed, and improved audio quality in certain sections.	**Technological Barriers:** Concerns about the rapid evolution of technology and regulations leading to outdated knowledge and the need for continuous learning and adaptation.	**Legal and Compliance Information:** How to handle billing and invoicing for telehealth services? Need for detailed guidance on GDPR compliance, data protection, and billing practices.
	**Legal and Compliance Information:**	**Perceived Bias:** Remarks about emphasis on certain	**Keeping Pace with Technological and Legal Changes:** What

	Provide more detailed guidance on GDPR compliance, data protection, and billing practices, especially concerning tools usable in their regions (e.g., Austria, Germany, Switzerland).	companies or products, suggesting a need for a more balanced presentation.	mechanisms can be put in place to ensure practitioners remain informed about the latest developments in telehealth?
	**Software Recommendations:** Participants sought specific, GDPR-compliant telehealth platforms suitable for private practitioners and non-insurance-based services.	**Language Use:** The use of gender-inclusive language was challenging for some, making it harder to follow the content.	**Interdisciplinary Collaboration Opportunities:** How can interprofessional learning and collaboration in telehealth training be facilitated to benefit from the experiences and insights of other professions?
		**Applicability:** Some felt telehealth might not be suitable for their patient demographics, such as elderly or less tech-savvy clients. Concerns that telehealth cannot replace the empathy and personal connection of in-person care.	**Role of Professional Associations:** What role can professional associations play in providing resources. Advocacy, and support for telehealth adoption among their members?
		**E-Learning Technical Limitations:** The inability to re-watch videos or access materials after completion was seen as a limitation, affecting the ability to reinforce learning.	**Support from Professional Associations:** Are there designated contact persons or resources within professional associations to assist with telehealth implementation? How can practitioners access ongoing support?
			**Addressing Technological Barriers for Patients:** How can healthcare professionals support

		patients, especially older adults or those with disabilities in using telehealth technologies to ensure services remain inclusive?
